# Relaxation of Adaptive Evolution during the HIV-1 Infection Owing to Reduction of CD4+ T Cell Counts

**DOI:** 10.1371/journal.pone.0039776

**Published:** 2012-06-29

**Authors:** Élcio Leal, Jorge Casseb, Michael Hendry, Michael P. Busch, Ricardo Sobhie Diaz

**Affiliations:** 1 Biotechnology Institute, Federal University of Pará, Belém, Brazil; 2 Institute of Tropical Medicine of Sao Paulo, University of São Paulo, São Paulo, SP, Brazil; 3 Centers for Disease Control and Prevention (CDC), Laboratory Branch, Atlanta, United States of America; 4 Dept. of Laboratory Medicine, University of California San Francisco; Blood Systems Research Institute; Blood Systems, Inc. San Francisco, United States of America; 5 Federal University of São Paulo, São Paulo, Brazil; Faculty of Medicine Tel Aviv University, Israel

## Abstract

**Background:**

The first stages of HIV-1 infection are essential to establish the diversity of virus population within host. It has been suggested that adaptation to host cells and antibody evasion are the leading forces driving HIV evolution at the initial stages of AIDS infection. In order to gain more insights on adaptive HIV-1 evolution, the genetic diversity was evaluated during the infection time in individuals contaminated by the same viral source in an epidemic cluster. Multiple sequences of V3 loop region of the HIV-1 were serially sampled from four individuals: comprising a single blood donor, two blood recipients, and another sexually infected by one of the blood recipients. The diversity of the viral population within each host was analyzed independently in distinct time points during HIV-1 infection.

**Results:**

Phylogenetic analysis identified multiple HIV-1 variants transmitted through blood transfusion but the establishing of new infections was initiated by a limited number of viruses. Positive selection (*d_N_/d_S_*>1) was detected in the viruses within each host in all time points. In the intra-host viruses of the blood donor and of one blood recipient, X4 variants appeared respectively in 1993 and 1989. In both patients X4 variants never reached high frequencies during infection time. The recipient, who X4 variants appeared, developed AIDS but kept narrow and constant immune response against HIV-1 during the infection time.

**Conclusion:**

Slowing rates of adaptive evolution and increasing diversity in HIV-1 are consequences of the CD4+ T cells depletion. The dynamic of R5 to X4 shift is not associated with the initial amplitude of humoral immune response or intensity of positive selection.

## Introduction

Intra-host HIV-1 diversity is highly affected by the intensity of the antibody response against viral epitopes in the initial stages of infection [1], [2], [3], [4]. In addition, over the HIV-1 infection time, the intensity of positive selection tends to decline [4] and the rate of synonymous mutations usually increases [5]. It has been suggested that selective pressure in HIV-1 is a direct function of CD4+ T cells levels [6]. Additionally, the affinity of the virus by host cells is equally important to determine rates of positive and purifying selection in V3 loop of HIV-1 [7]. However other features of the virus life cycle may equally affect the rate of adaptive evolution of HIV-1 [8].

During the progress of HIV-1 infection in nearly half of individuals the virus changes its chemokine receptor usage [9], [10]. Usually, in the early phase of infection the HIV-1 has a tropism for CCR5 (R5 variants), in the late phase of the infection viruses (X4 variants) that preferentially use CXC4 emerge. At the late phase of HIV-1 infection, the intra-host viral population may be composed exclusively by X4 variants, by variants able to use both coreceptor (R5X4) or the viral population can be represent by similar proportions of R5 and X4 variants [11], [12]. In addition, R5 and X4 viruses can even recombine in the course of intra-host HIV-1 infection [13]. These variants have distinct properties the emergence of X4 variants is usually associated with a decline of CD4 cells and rapid disease progression [10], [14], [15]. Therefore, it has been suggested that X4 viruses might be more virulent than R5 viruses. R5 can be isolated preferentially from CD4+ memory T cells and X4 can be isolated from both memory and naïve cells [16], [17]. However, the reason for the change in the coreceptor usage and why it is restricted to a few patients remains uncertain.

It has been suggested that R5 variants may have a selective advantage because it has a higher replication rate in memory cells, compared with naïve cells [18], [19]. In addition, in resting CD4+ T cells R5 variants, but not X4 variants, induce expression of genes involved in cell proliferation, this might facilitate the replication of R5 variants [20]. These findings might explain the predominance of R5 in the early infection, but not the later emergence of X4 variants. Recently, it has been shown that the turnover between naïve and memory cells might explain the switch from R5-to-X4 variants in late infection [21].

There are few studies that focused specifically on immune response and the impact of R5-to-X4 shift evolution from the same viral source in epidemic linked hosts. Thus to gain further insights into the evolutionary HIV-1 within hosts, we evaluated sequences from an epidemic cluster where the same viral source from a HIV-infected donor contaminated two blood recipients. One of the recipients infected, through sexual intercourse, her male partner shortly after she received the contaminated blood transfusion. In the blood donor and in one recipient, X4 viruses emerged independently and the outcome of the virus dynamics in each individual was also distinct. A detailed Bayesian analysis was performed in molecular clones generated in distinct time points from each individual of the epidemic cluster.

## Results

### Pairwise genetic diversity

**Figure 1:**
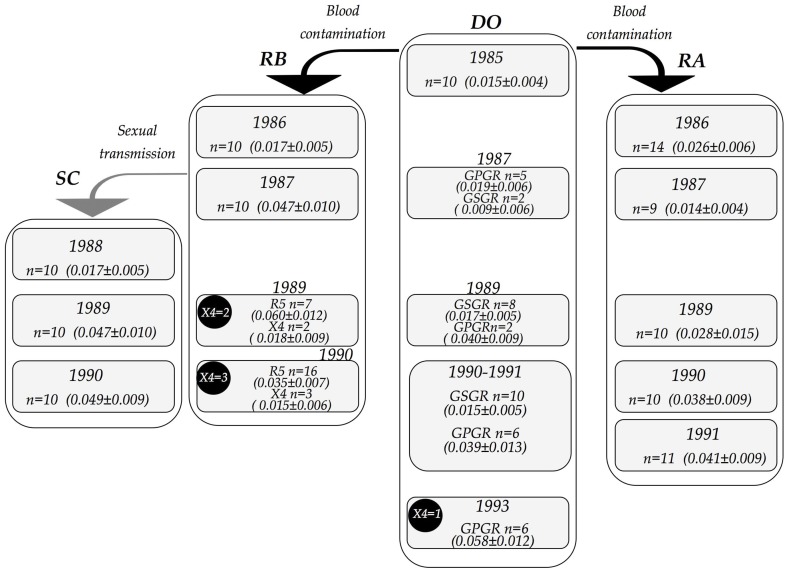
Schematic representation of the blood-transmission cluster. Each column represents all samples of one patient. DO: donor; RA: recipient A; RB: recipient B; SC: sexual partner of the RB. Gray rectangles indicate each time-point with sampling date, number of clones generated and the mean of the pairwise diversity plus standard error. Filled circles indicate number of X4-variant sequences. Arrows indicate the date and the transmission route.

Figure 1 shows the transmission cluster and genetic diversity at distinct time points according to the sampling schedule of the study. The blood donor (DO), a 30 years old male, evolved without apparent clinical or immunological progression with a stable CD4 in a follow up from 1985 to 1993. Interestingly, in 1987 a minority variant with GSGR tetramer at the tip of V3 loop was detected in the DO sequences. In 1989 this GSGR variant was majority (n = 8) compared with the previous GPGR variant (n = 2). Between 1990 and 1991 a mix of GSGR (n = 10) and GPGR (n = 6) variants was observed. Notably, in 1993 GSGR variants were no longer detected in DO samples, concomitantly, in this year we identified one X4 strain (filled circles in the Figure 1). Genetic diversity was evaluated using pairwise distances. From 1985 to 1993 the mean diversity of DO sequences increased from 0.015±0.004 to 0.058±0.012, respectively. Since samples from patient DO were composed by two distinct variants (*viz*., GSGR and GPGR) we analyzed separately the genetic diversity of each one. In 1987 two samples of GSGR variant was detected and their genetic diversity was 0.009±0.0046, whereas the diversity of GPGR variants at this year was 0.019±0.006. In 1989 GSGR variants predominated in the samples (8 out 10 sequence) and had mean diversity of 0.017±0.004. Particularly, although in minority (n = 2) GPGR variants presented a high mean genetic diversity (0.040±0.009). On the other hand, between 1990 and 1991 there was equilibrium of GSGR and GPGR sequences in the DO samples. The genetic diversity of GSGR variants was maintained at lower level of 0.015±0.004 while the mean genetic diversity of GPGR remained high at mean of 0.039±0.013. In 1993 only GPGR were detected and the mean genetic diversity equally increased to 0.058±0.012. Although GSGR prevailed in 1989 its genetic diversity was always at lower levels in the interval between 1987 and 1991 (ranging from 0.009 to 0.015). In opposition, GPGR variants presented a constant increasing diversity, even when it in minority proportion (2 out 10) in 1989 (0.040±0.009). Notably, the fluctuation in the proportions of GSGR and GPGR variants in the viral population of DO patient apparently were not correlated with the genetic diversity, since the less divergent GSGR viruses out-competed the GPGR lineage between 1989 and 1991.

The first recipient analyzed (RA) was a 57-year-old male that received red blood cells from blood donor due to a bleeding duodenal ulcer and also evolved with a stable CD4 without a detectable emergency of X4 related strains from 1986 to 1991. Sequences from RA individual obtained at the start of infection in 1986, revealed high genetic diversity with mean of 0.026±0.006. In this year, two phylogenetically distinct groups of sequences were detected (see phylogenetic analysis), each one with similarly high genetic diversity. The mean genetic diversity of 0.026±0.009 was detected in the first and the mean of 0.023±0.005 was detected in the second group. However, in 1987 the mean genetic diversity decreased to 0.014±0.004. Subsequently, in the following years (1989 to 1991) HIV samples from patient RA increased continuously its genetic diversity (Figure 1).

The second recipient analyzed (RB) was a female contaminated by the same viral source (same blood donation) that was inoculated in the recipient RA. RB was transfused in January 1985 at the age of 20 year old with platelets due to a *post-partum* bleeding. The mean genetic diversity of viral sequences from RB increased continuously from 1986 to 1989, respectively 0.017±0.005 to 0.060±0.012. Then it decreased slightly to 0.035±0.007 in 1990. Coincidently RB maintained high counts of CD4+ T cells after her infection in 1985 until 1989, then counts of CD4+ T cells decreased deeply and she evolved to AIDS and death in 1990 (levels of the CD4+ T cell counts are shown in the Figure S1). Notably, after X4-strains emerged in 1989 it persisted always as minority in the viral population of RB during all course of infection. In addition, pairwise distances of X4-strains sequences showed a limited degree of genetic diversity, with mean of 0.018±0.009 in 1989 and 0.015±0.006 in 1990. Interestingly, the emergence of X4-strains in the donor (DO) and recipient B (RB) coincided with the high peak of overall pairwise diversity.

The male partner of RB was infected sexually by her and evolved with a stable CD4 counts until 1990 with no detectable X4-strains sequences. The genetic diversity of viral population of the sexual partner (SC) was also limited in the start of infection in 1986 with mean of 0.017±0.005. Then the mean genetic diversity increased continuously until reach its high level of 0.049±0.009 in 1990 (Figure 1).

### Phylogenetic analysis

**Figure 2:**
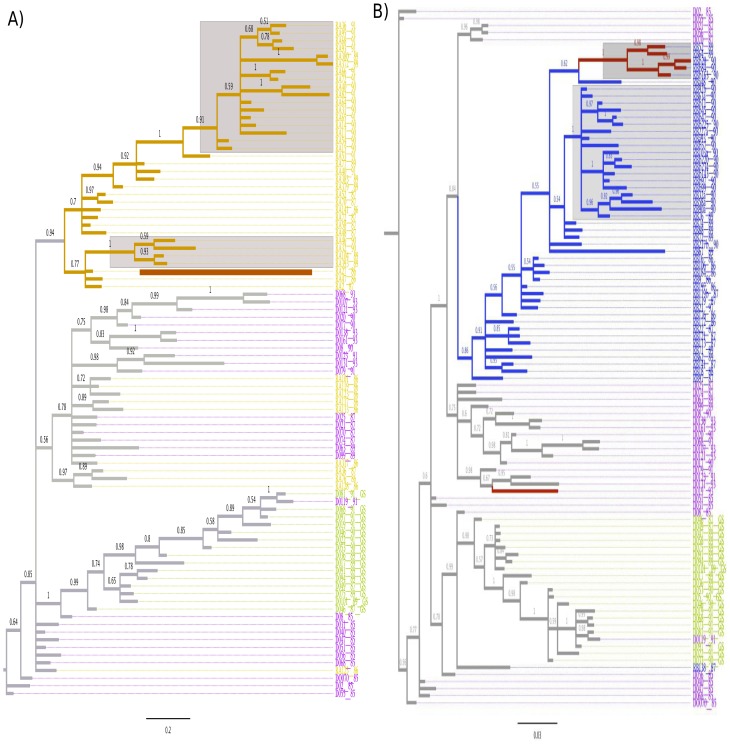
Maximum a posteriori tree of the blood donor and the recipients. This tree was constructed using molecular clones of the donor (DO) and the blood recipients. Sequences from all time points were included. The sampling time of clones are indicated in the sequences names (last two digits). Highlighted areas designate clones isolated the late stages of infection. Numbers above the branches indicate the Bayesian posteriori statistical support for the tree clades. A) The sequences of the donor DO are colored in magenta (GPGR variant) and in green (GSGR variants). The sequences of the recipient RA are colored in orange. Branches colored in orange indicate viruses that spread over the infection time in the RA. B) All clones generated from 1987 to 1990 from the HIV infection of the recipient RB were also included. The sequences of the recipient RB are colored in blue. The blue branches indicate viruses that spread over time in the RB. The red branches in the tree indicate the X4 variants.

Maximum posteriori (MP) trees were constructed initially to establish the relationship among the isolates of the donor and the blood recipients. All sequences obtained within a certain patient displayed an ordered pattern of time in the trees, according to their dates. Time ordered trees, thus provide an evidence for no recombination among variants within hosts. In addition, recombination was also verified by other methods [22]. In the figure 2A, the topology of the tree constructed with sequences of the donor DO and the recipient RA revealed that viruses from the donor (sequences indicated in magenta and green color) composed two distinct phylogenetic clusters. One cluster included variants having the GPGR motif at the V3 loop of the envelope gene. These variants probably derived from the founder viruses that infected the blood donor because isolates with GPGR were detected early in 1985. The other cluster included variants with the GSGR motif (green sequences in the Figure 2A) at the V3 loop and they emerged late in 1987 (we removed the second amino acid from the V3 loop tetramer). In addition, this tree also showed that the sequences isolated before 1990 from the recipient RA (indicated in orange color in the tree) intermingled with the sequences from the donor. While, those sequences isolated in 1990 and 1991 probably originated from same ancestors (highlighted in blue in the Figure 2A). Consequently, although multiple variants were transmitted to the blood recipient RA, quite few of them effectively contributed to establish the new infection.

Likewise, the MP tree constructed with molecular clones from the donor and the recipient RB (sequences indicated in blue color in the Figure 2B) indicated that multiple clones were transmitted through blood contamination. Particularly, the sequence RB138_87 is only RB isolate related with GSGR viruses from the donor. The tree also revealed that all clones generated from the blood recipient RB, with the exception of the sequence RB138_87, shared the same 1987 ancestor sequence. Therefore, the viral population of RB expanded probably from few variants. Notably, X4-variants (sequences indicated in red color in the Figure 2B) emerged independently in the donor in 1993 and in the recipient RB in 1989. Finally, the tree constructed to evaluate the dynamic of sexual transmission using sequences of recipient RB and sequences of the sexual partner (SC) revealed that the viral population of the patient SC is completely monophyletic (Figure S2).

### Pattern of N-linked glycosilation

The pattern of N-linked glycosylation is mediated by humoral immune escape antibody attack. There is a linear correlation between infection time and the amount of glycosylation in the V3 loop of HIV-1 [23]. It has been shown that in the first phase of HIV-1 infection the virus needs to bind and interact with cellular receptors, then it has to reduce the number of glycosilated sites. Conversely, HIV-1 evades antibody attack by the acquisition of mutations at N-linked glycosylation sites. These mutations permit the HIV to avoid binding of neutralizing antibodies to the viral surface proteins, consequently virus to escape from the humoral immune surveillance, this mechanism is better know as the “glycan-shield” [24]. We investigated the proportion of N-linked glycosylation sites and found that in general the amount of glycosylated sites increased in viruses isolated in the late stages of HIV infection (clusters highlighted in the Figure S3). The exceptions were X4 viruses in the recipient RB (red sequences in the Figure S3) and GSGR viruses of the donor DO (highlighted in cyan). Sites glycosylated in the initial viral sequences usually remained unchanged during the infection, and new mutations were not necessarily located in the same sites in all patients (number within parenthesis in the Figure S3). The most radical changes in the location of glycosylation sites were observed in the intra-host viruses of the recipient RB (sequences depicted in blue and red colors in the Figure S3).

Details of the CD4 levels (Figure S1), antibody affinity by peptides (Figure S4), neutralization profile of each patient (Figure S5) and virus load (Figure S6) can be found at supporting information.

### Selective Regimen

In order to gain deeper insights into the selective regimen of HIV-1 during natural infection, a systematic codon-based analysis was performed. According to the estimated likelihoods of each model, the hypothesis of positive selection (which is based on the likelihood of the M3 model that has three categories *d_N_/d_S_* (ω_0_, ω_1_ and ω_2_)) was widely accepted, with the exception to the data set RB (1989–1990) (Table 1). The estimated parameters for the DO clones sampled between 1985 and 1993 indicated that the overall percentage of positively selected sites was 10% and the estimated mean ω value was 7.4. The M3 model also detected the codons 5, 33, 44 and 70 with high posterior probability (≥0.99) of positive selection in the fragment of *env* gene of HIV-1. Since the viral population of recipient DO was composed by two distinct variants (see the phylogenetic analysis above), they were analyzed separately. The analysis of the dataset with molecular clones of the GSGR variant sampled between 1987 and 1991 showed 6% of sites positively selected with a mean ω value of 13, and the codon 44 was detected with high posterior probability (Table 1). The other dataset with sequences of GPGR variants sampled between 1987 and 1991 detected the same proportion of 6% of sites positively selected and the mean ω value was also 13, the codons 33, 44 and 70 were detected with high posterior probability of been under positive selection. Interestingly, codons 33 and 44 correspond respectively to codons 11 and 25 of V3 loop; which are related with R5-to-X4 change in HIV-1.

**Table 1:** Maximum likelihood estimates of *d_N_* and *d_S_* using codon-based models.

Model *ℓ = likelihood*	Parameters estimated	*2Δℓ* 1	Positively selected sites#
***DO GP-variants (1985–1993)*** ∶ ** (n = 42, l = 228)**			
M_0_ (one-ratio) ***ℓ_0_*** = −837.29	*ω* = 0.734	*87.419*	*Not allowed*
M_3_ (discrete)* ***ℓ_1_*** = −793.58	*P*_0_ = 0.697 *p*_1_ = 0.201 *p*_2_ = 0.102 *ω*_0_ = 0.174 *ω*_1_ = 1.211 *ω*_2_ = 7.396		*5, 33, 44, 70*
***DO GS-variants (1987–1991) (n = 15, l = 228)***			
M_0_ (one-ratio) ***ℓ_0_*** = −448.12	*ω* = 1.342	*11.830*	*Not allowed*
M_3_ (discrete)* ***ℓ_1_*** = −442.20	*P*_0_ = 0.517 *p*_1_ = 0.422 *p*_2_ = 0.061 *ω*_0_ = 0 *ω*_1_ = 2.187 *ω*_2_ = 13.079		*44*
***DO GP-variants (1987–1991) (n = 14, l = 228)***			
M_0_ (one-ratio) ***ℓ_0_*** = −590.25	*ω* = 0.967	*61.264*	*Not allowed*
M_3_ (discrete)* ***ℓ_1_*** = −559.61	*P*_0_ = 0.831 *p*_1_ = 0.102 *p*_2_ = 0.067 *ω*_0_ = 0.235 *ω*_1_ = 1.173 *ω*_2_ = 13.112		*33, 44, 70*
***RA (1986–1991) (n = 46, l = 228)***			
M_0_ (one-ratio) ***ℓ_0_*** = −954.51	*ω* = 0.713	*83.902*	*Not allowed*
M_3_ (discrete)* ***ℓ_1_*** = −912.53	*P*_0_ = 0.653 *p*_1_ = 0.311 *p*_2_ = 0.036 *ω*_0_ = 0.057 *ω*_1_ = 1.896 *ω*_2_ = 5.537		*14*
***RB (1986–1990)*** ∶ ** (n = 58, l = 228)**			
M_0_ (one-ratio) ***ℓ_0_*** = −947.89	*ω* = 0.922	*123.677*	*Not allowed*
M_3_ (discrete)* ***ℓ_1_*** = −886.05	*P*_0_ = 0.631 *p*_1_ = 0.266 *p*_2_ = 0.102 *ω*_0_ = 0.141 *ω*_1_ = 1.490 *ω*_2_ = 10.723		*5, 7, 44*
***RB (1986–1987) (n = 21, l = 228)***			
M_0_ (one-ratio) ***ℓ_0_*** = −583.90	*ω* = 0.525	*44.913*	*Not allowed*
M_3_ (discrete)* ***ℓ_1_*** = −559.45	*P*_0_ = 0.315 *p*_1_ = 0.570 *p*_2_ = 0.116 *ω*_0_ = 0.000 *ω*_1_ = 0.326 *ω*_2_ = 6.511		*5, 30, 44*
***RB (1989–1990)*** ∶ ** (n = 23, l = 228)**			
M_0_ (one-ratio) ***ℓ_0_*** = −772.86	*ω* = 0.718	*29.160*	*Not allowed*
M_2_ (selection)$ ***ℓ_1_*** = −758.28	*P*_0_ = 0.577 *p*_1_ = 0.159 *p*_2_ = 0.263 *ω*_0_ = 0.127 *ω*_1_ = 1.000 *ω*_2_ = 1.977		*none*
***SC (1988–1990) (n = 32, l = 228)***			
M_0_ (one-ratio) ***ℓ_0_*** = −796.11	*ω* = 1.651	*143.48*	*Not allowed*
M_3_ (discrete)* ***ℓ_1_*** = −759.40	*P*_0_ = 0.550 *p*_1_ = 0.321 *p*_2_ = 0.129 *ω*_0_ = 0.002 *ω*_1_ = 2.292 *ω*_2_ = 9.076		*15, 44, 48, 60, 70*

1) Assuming that M_0_ is true null hypothesis (*Η*_0_) with *p*_0_ parameters and that M_3_ is the alternative hypothesis (*Η*_1_) with *p*_1_ parameters. The log likelihood difference ***2Δℓ = 2(ℓ_1_−ℓ_0_)*** follows asymptotically a χ^2^ distribution with (*p*_1_−*p*_0_) degrees of freedom.

*) Best-fitted model according to the likelihood ratio test (LRT) between of M_0_ vs. M_3_ with 4 degrees of freedom.

$) Best-fitted model according to the likelihood ratio test (LRT) between of M_2_ vs. M_3_ with 1 degree of freedom.

#) Positively selected sites were detected according to the Naive Empirical Bayes method with p≥0.99. Sites 33 and 44 (underlined) correspond respectively to sites 11 and 25 of V3 loop; which are related with R5-to-X4 change.

∶) This result was obtained using unique sequences and X4-strains because exclusion of them did not affect on the model preference or the parameter values.

The analysis of recipients' sequences also revealed the presence of positive selection in diversification of HIV-1. For RA dataset we found 4% of codons under positive selection with mean ω value of 5.5 (Table 1). In this case, only the codon 14 was detected with high posterior probability of positive selection. Similarly, the proportion of positively selected sites was 5.5% with a mean ω value of 6.9 for the dataset with all sequences of RB. Additionally, the M3 model detected the codons 5, 7 and 44 with high posterior probability of positive selection for RB sequences. Further subdivisions were also made with the sequences of RB (Table 1) where two time intervals were analyzed. First, sequences sampled between 1986 and 1987 were analyzed. The result showed 12% of positively selected codons with a mean ω of 6.5 and the sites 5, 30 and 44 were detected with high posterior probability. Second, another dataset with RB sequences sampled between 1989 and 1990 was also studied. In this case, model M2 (selection) was the best fitted and indicated that 26% of sites were positively selected with a mean ω of 1.9, although the high posterior probability indicated there are no codons under positive selection.

**Figure 3:**
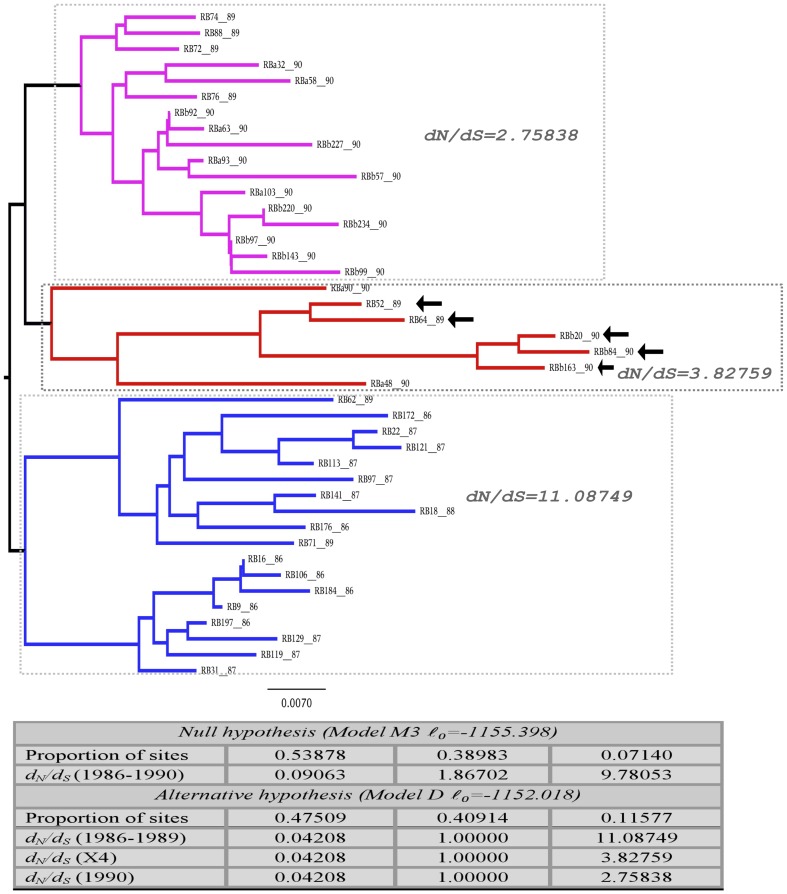
Adaptive evolution during the natural HIV-1 infection. A maximum likelihood tree was inferred with unique V3 region sequences of the blood recipient RB. Branch-site model was used to evaluate if specific regions (clusters corresponding to distinct chronological times) of the tree were under distinct selective pressures. The tree depicts selective pressures (dN/dS) in distinct moments (years) of HIV-1 infection. The ratio dN/dS was estimated in isolates sampled from 1986 to 1989 (blue branches), then in isolates from 1989 to 1990 (pink branches) and finally in the cluster where X4 viruses appeared (red branches, X4 variants are indicated by arrows within this cluster). Dotted lines delineate each group of sequences where (dN/dS) was estimated. The last two digits in each name designate the year that a specific sequence was sampled. The panel in the bottom of the figure shows the estimated parameters of model M3 (null hypothesis) and model D (alternative hypothesis) (see the manuscript for details).

To better explore the observed reduction in the strength of positive selection of RB viruses sampled between 1989 and 1990, we used a branch-site model (see model D in methods for details). This model permits to investigate if a specific part of a phylogenetic tree has ω values distinct than that estimated to other branches of the tree [25]. We used unique sequences sampled from RB between 1986 and 1990 and investigated if some branches (depicted in colors in the Figure 3) would have sites with distinct selective pressures. Results indicate that sequences isolated in 1990 evolve distinctively than those sequences isolated before this time point according to LRT (χ^2^ = 6.759926, *p* = 0.009). Overall, the model detected 47% of sites under strong purifying selection (ω = 0.042) and 41% of neutral (ω = 1). In addition, there is a set of codons (≅12%) under strong positive selection (ω = 11.0875) in sequences isolated between 1986 and 1989. This set of codons experienced reductions in the intensity of positive selection to ω = 2.758 in sequences isolated in 1990 and to ω = 3.827 in the cluster where X4 viruses emerged. This results indicate that reductions in the intensity of positive selection is not restricted to X4 sequences rather it is an overall feature of all sequences isolated after 1990.

Lastly, for the dataset that included all sequences of the sexual partner SC the model M3 identified 13% of sites under positive selection with mean ω of 9.0; the codons 15, 44, 48, 60 and 70 were detected high posterior probability of positive selection (Table 1). It is noteworthy that codon 44 is related R5-to-X4 phenotype shift in HIV-1, this codon was under positive selection in individuals DO and RB in both X4-variants were detected as minor variant in the intra-host viral population. Codon 44 was also under positive selection in the intra-host viral population of individual SC, although X4-varints were not detected in this patient.

## Discussion

Since the first stages of HIV-1 infection might be crucial for the characterization of viral diversity and the antibody response against viral epitopes might be the leading force driving HIV diversification in the initial phase of infection [1], [2], [3]. Thus, the observation that monoclonal antibodies preferentially neutralize X4 variants [26] might explain the initial advantage of R5 viruses. However, it has been shown that recent emerged X4 variants are more sensitive to antibody neutralization than late emerged X4 variants [27]. Consequently, X4 variants might effectively adapt to escape from antibody neutralization and establish as new lineages in the late stages of HIV infection. In addition, the HLA profile would be insightful to the further understand the pattern of selective pressure in the quasispecies within each patient, unfortunately this information not available in our study. Of note, X4 viruses observed within the recipient RB presented reduced number of glycosylation sites compared to other variants, thus suggesting that to infect host cell newly emerged X4 variants lessen the protection of the “glycan shield”. X4 viruses isolated in late stages of RB infection maintained low levels of glycosylation, making them more vulnerable to antibody neutralization. Furthermore, it has been suggested that structural features of V3 loop might also constraint the R5-to-X4 shift within-hosts [28]. Thus, the background of the virus that initiates the infection might also contribute to the appearance and establishing of X4 variants.

On the other hand, considering that HIV has extremely high rates of adaptive mutations of HIV [29] and the emergence of X4 variants is associated with the acquisition of a reduced positively charged amino acids in V3 loop [28], [30], [31]. Then, the selective pressure imposed solely by antibody neutralization might partially explain the late emergence of X4 viruses, but not the replacement of R5 variants or the decline of CD4 levels associated with of X4 viruses. Our results showed relaxation of positive selection, in the patient RB, occurred equally in X4 and R5 viruses. Consequently, there is no evidence of distinct adaptive evolution between these variants that would favor X4 variants to predominate in the infection.

Our results also showed that appearance of distinct HIV viruses occurs even when the overall diversity is low (*i.e*, GSGR variants). Thus, the genetic divergence (measured by the pairwise diversity) in the epidemic cluster has no relation to the dynamic process of lineages replacement. The intra-host HIV population of the donor DO was characterized by the emergence of GSGR variant two years after initial infection. This less divergent strain was the main variant between 1989 and 1991, while the GPGR strain (that initiated the infection and was highly divergent) reappeared and was the only variant detected in 1993. Although selective pressure has been pervasive, including in V3 loop sites associated with R5-to-X4 shift (Table 1), the pattern of X4 emergence was quite distinct among the individuals of the epidemic cluster. Interestingly, X4-strains were not detected in the viral population of the recipient RA and in the sexual partner SC during the study. These results suggest that although the genetic background of the virus is important to determine the emergence of X4 strains, the cellular environment of hosts might be the key factor to control the timing of R5-to-X4 shift. Interestingly, the emergence of X4 strains in the donor (DO) and recipient B (RB) coincided with the high peak of overall pairwise diversity.

Although the patient RA was treated with zidovudine since 1988 and the patient RB started with zidovudine monotherapy in 1989, the pairwise diversity of HIV was not affected. Figure 1 shows that viruses within the patient RA presented low diversity prior to the therapy, whereas viral diversity in the patient RB continuously increased since 1987.

Detailed analysis, performed in an known HIV transmission chain showed that trees inferred using the *pol* gene were unable to rescue the real evolutionary history of the virus because the appearance of convergent drug-associated mutations. On the other hand, trees constructed using the *env* gene were not affected by reverse-transcriptase and protease inhibitors [32]. The effect of antiretroviral therapy on the HIV diversity is highly specific: antiviral drug will increase the adaptive value of mutations that confer resistance to the virus. In fact, codons associated with drug resistance in the RT gene of HIV evolve under purifying selection in absence of therapy and shift to positive selection when therapy initiates, while the overall intensity of selective pressure remained unchanged [33].

It has been observed that selective pressure declines over time in the intra-host HIV-1 population [4], [5]. Previously, we found that intensity of positive selection in HIV-1 is directly related with the number of CD4+ cell [6]. Lemey et al. 2007, noticed an increase in the rate of neutral mutations associated with disease progression and suggested it was affected by the rate of HIV-1 replication (viral generation time). It is noteworthy that the increasing diversity detected in 1989 in the recipient RB coincides with the severe decline of CD4+ T cell counts. This reduction of CD4 cells might indicate loss of integrity of the immune system to mount response directed to new viral epitopes. In this regard is tempting to suppose that relaxation of immune pressure creates an ideal cellular environment that increases the changes of newly emerged variants not to be eliminated from intra-host viral population. The replication rate observed in the patient RB coincide with the possibility that elevated rate of naïve cells divisions facilitate X4 overgrowth [21] and provide further support to the importance of host cellular environment to the dynamics of R5-to-X4 shift. Indeed, random processes are likely to define HIV variants that will prevail in the viral population of recently infected individuals [8].

Taken together all these features, it is quite clear that HIV-1 evolution within host is a complex process that might involves subtle equilibrium between adaptation to the cellular environment, escape of the immune response and perhaps random fixation of variants. By using pairwise distances and branch-site models we showed that HIV-1 diversity in the late stages of infection increases and positive selection is relaxed. It is likely that the observed increase in diversity occurred due to the accumulation of neutral mutations. Although the decline of positive selection and the increase in of genetic diversity observed in the late stages of HIV-1 infection have been associated with humoral immune response [1], [4], [5]. Here we show varying levels of humoral immune response over infection time had little impact to HIV diversity, however reduction of CD4+ T cells counts is the main cause of the low intensity of positive selection.

## Materials and Methods

### Ethics statement

This study was approved by the Ethics Committee of the Federal University of São Paulo and by the Brazilian Ministry Health; all biological samples were obtained in full accordance with signed informed consent forms.

### Sequences and data set compilation

All sequences used in this study were generated in a previous study [34]. Briefly, viral RNA from plasma was used to generate molecular clones by limiting dilution PCR from viruses isolated from distinct time-point, these sequences were from a 231-bp fragment of *envelope (env)* gene from HIV-1 subtype B. A total of 200 clones (156 unique sequences) were generated, they were from an epidemic cluster composed by the donor (DO); two blood recipients (RA, RB) and one individual infected by one of the recipient by sexual contact (SC). These individuals were not exposed to antiviral drug during the time interval of the study, thus the mutation pattern of the sequences represent the natural evolution of HIV in its host. The transition route and the samplings time are schematically represented in the figure 1. The alignment contained all sequences used can be obtained at http://www.biotorrents.net/.

### Sequence alignment

The sequences were initially aligned using the ClustalX program [35], then sites with deletions and insertions were then excluded in order to preserve the reading frames of the genes. After this editing process, the sequences were manually aligned using the SE-AL program, version 2.0 (Department of Zoology, Oxford University; http://evolve.zoo.ox.ac.uk/software/). To compute pairwise distances we used the Kimura two-parameters model, the estimates were performed using the Mega software version 4.0 [36]

### Proportion of N-linked glycosylation sites

The N-linked glycosylation sites were determined by identifying the *NX[ST]Y* pattern (where X can be any amino acid). Amino acids, such as proline, in the X or Y position can be important determinants of N-linked glycosylation efficiency [37]. The analyses were performed using the web interface available at http://www.hiv.lanl.gov/content/hiv-db/GLYCOSITE/glycosite.html.

### Phylogenetic inference

To construct the trees we used Bayesian methods, assuming GTR and a gamma correction model, as is implemented in the MrBayes program, version 3.1.2 [38]. Two independent Markov chain Monte Carlo (MCMC) processes were run for 2×10^7^ generations with the initial 10% of each run discarded as burn-in. The convergence of chains was evaluated using the TRACER software, version 1.2 (http://beast.bio.ed.ac.uk/), and runs were accepted when all parameters presented the effective sample size number (ESS) greater than 100. Maximum posteriori (MP) trees were show and edited using the FigTree software (http://tree.bio.ed.ac.uk/software/figtree/).

### Prediction of coreceptor usage

In addition to the presence of basic amino acids at sites 11 and 25 of the V3 loop (11/25 rule), the position-specific scoring matrices (PSSM) method was also used in our analyses [39]. The PSSM detects nonrandom distributions of amino acids in a set of sequences that may be associated with some empirically determined property (for example, coreceptor usage) and constructs a matrix. The matrix is then used to compare the amino acids of query sequences and PSSM assigns scores to each query sequence. Consequently, these scores can be used to predict the likelihood of a query sequence having the property of interest. We predicted the coreceptor usage of our sequences using a PSSM matrix (x4r5) constructed with the V3 region of HIV-1 subtype B with a known X4 or R5 phenotype (http://mullinslab.microbiol.washington.edu/computing/pssm/). Results can be interpreted as follow; high scoring sequences are more likely to use the CXCR4 coreceptor, while low scoring sequences are more likely to use the CCR5 coreceptor.

### Detection of selective pressure

Selective pressures were analyzed using an approach that estimates the number of *d_N_* and *d_S_* at all sites in the sequence alignments. The method compares the fit to the data of various models of codon evolution, which differ in the distribution of *d_N_/d_S_* (ω) among codons and takes into account the phylogenetic relationships of the sequences [40].

The model 0 (M0: one-ratio) assumes a single ω for all codons in the alignment and hence is the simplest model specified.

The model 1 (M1: neutral) allows different proportions of conserved (ω = 0) and neutral codons (ω = 1), both estimated from the data. The model 2 (M2: selection) has an additional class of codons with its ω ratio (which can be >1) estimated from the data. The model 3 (M3: discrete) also allows positive selection by incorporating three categories of codons with the ω value at each estimated from the data. Nested models can be compared using a standard likelihood ratio test (LRT). Significant evidence for positive selection is provided if M2, or more normally M3, significantly reject the null hypothesis M0 and M1, and if the favored models contain a class of codons where ω>1. We also used an extension of M3 to investigate if there are sites evolving under different selective pressure, this model allow to detected sites not necessarily under positive selection [25]. This model (model D with k = 3) was used to determine if a specific group of sequences (*i.e.*, sequences of recipient RB sampled in 1990) was subjected to a distinct selective regimen in a phylogenetic tree constructed with sequences sampled over the HIV-1 infection time in individual. Since model D is nested to M3, we used LRT and M3 as the null hypothesis assuming one degree of freedom. All these models are implemented in the CODEML program from the PAML v. 3.14 package (http://abacus.gene.ucl.ac.uk/software/paml.html). To the positive selection estimate molecular clones of each patient were analyzed separately. Additional subdivisions of the intra-host molecular clones were also made in order to obtain more detailed information on a particular time interval. For example, sequences of the donor DO were first analyzed in a time interval between 1985 and 1993. Then these sequences were subdivided into two other datasets; one composed by HIV-1 variants with the GPGR tetramer at the V3 loop of *env* gene and other composed by variants with GSGR tetramer. These two datasets included clones sampled between 1987 and 1991 because GSGR viruses emerged after 1987 (see results).

## Supporting Information

Figure S1 **CD4+ T cell levels during HIV-1 infection.** The numbers of CD4+ T cells per mm3 of each time point of the infection are represented by colored lines. Each line represents one individual.
(TIFF)

Figure S2 **Maximum a posteriori tree of the recipient RB and the patient SC.** This tree was constructed using molecular clones of the recipient RB and her sexual partner SC. Sequences from all time points were included. The sampling time of clones are indicated in the sequences names (last two digits). Numbers above the branches indicate the Bayesian posteriori statistical support for the tree clades. A) The sequences of the recipient RB are indicated in blue and in red colors (X4 variants). The sequences of the patient SC are colored in orange. The tree was rooted at the midpoint.
(TIFF)

Figure S3 **Glycosylation pattern of the V3 loop of HIV-1.** Numbers above branches indicate glycosylated sites in the V3 region. The tree shows clones of within-host sequences of the blood donor DO (depicted in pink color) and the recipients RA (depicted in green) and RB (depicted in blue and red color for X4 isolates). Highlighted areas represent clusters of isolates obtained from the last time points in each individual. The statistical support of the tree is indicated by the colors of branches in a gradient scale from yellow to red, respectively indicating posterior probability of 0.5 to 0.99. Some branches were collapsed to facilitate visualization.
(TIFF)

Figure S4 **Antibody affinity by autologous synthetic peptides.** The humoral immunity of each patient was measured by the affinity of their antibody to recognize peptides based on consensus sequences of virus isolated in distinct time points. Affinity was measured independently in each individual in distinct time points.
(TIFF)

Figure S5 **Neutralization of the heterologous MN strain of HIV-1.** Humoral immune response against the MN strain of HIV-1 was measured in the plasma of patients during the over the infection time. Each line represent one individual and points are the measured immune response detected in each year (x-axis).
(TIFF)

Figure S6 **Virus load of each patient during infection time.** The x-axis represents the sampling time of the study. Y-axis depicts the amount of viruses measured in RNA copies per ml of plasma.
(TIFF)
