# Supplementary figures and images for: Relaxation of Adaptive Evolution during the HIV-1 Infection Owing to Reduction of CD4+ T Cell Counts
Source: PLoS One. 2012 Jun 29;7(6):e39776. doi: 10.1371/journal.pone.0039776 (PMC3387245; doi:10.1371/journal.pone.0039776)

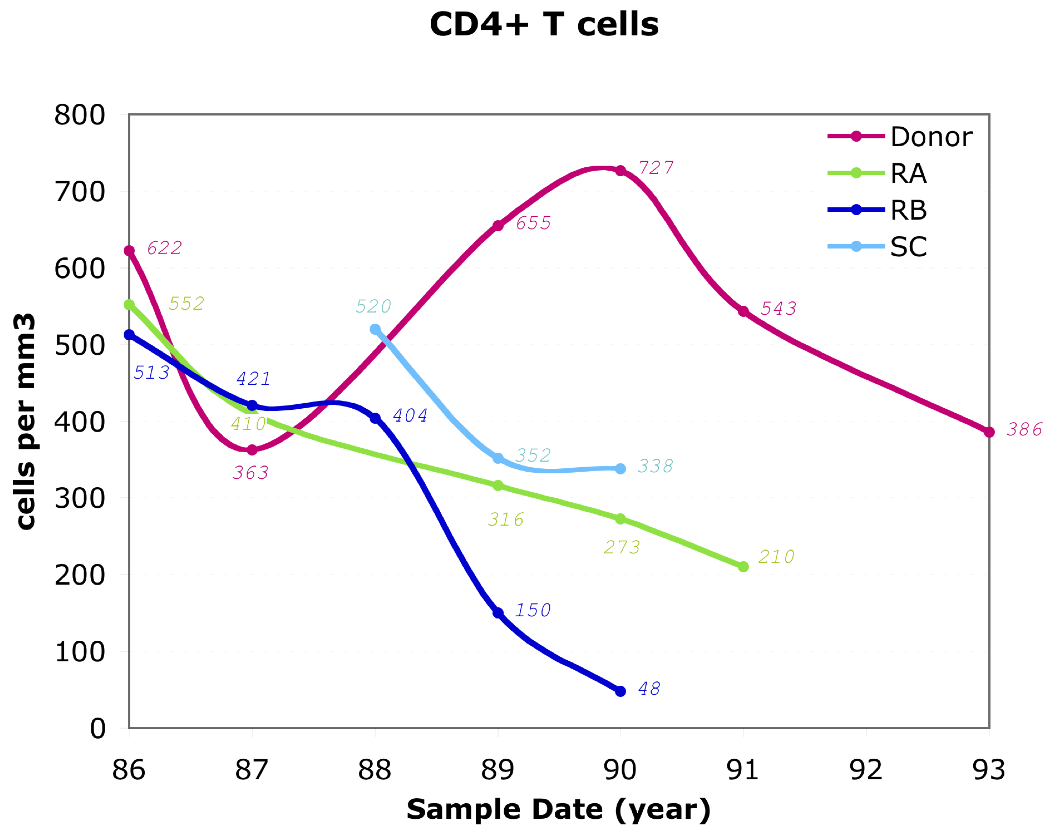

Supplement: Figure S1 — CD4+ T cell levels during HIV-1 infection. The numbers of CD4+ T cells per mm3 of each time point of the infection are represented by colored lines. Each line represents one individual. (TIFF) [file pone.0039776.s001.tiff]

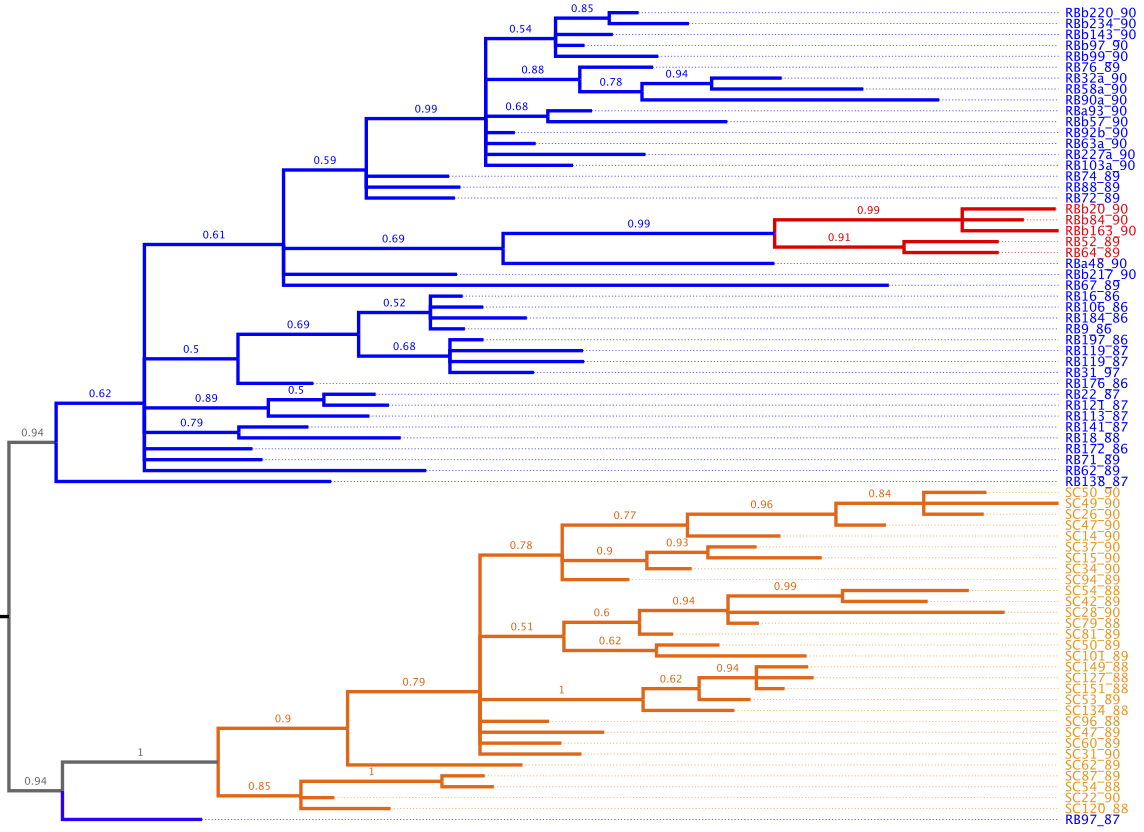

Supplement: Figure S2 — Maximum a posteriori tree of the recipient RB and the patient SC. This tree was constructed using molecular clones of the recipient RB and her sexual partner SC. Sequences from all time points were included. The sampling time of clones are indicated in the sequences names (last two digits). Numbers above the branches indicate the Bayesian posteriori statistical support for the tree clades. A) The sequences of the recipient RB are indicated in blue and in red colors (X4 variants). The sequences of the patient SC are colored in orange. The tree was rooted at the midpoint. (TIFF) [file pone.0039776.s002.tiff]

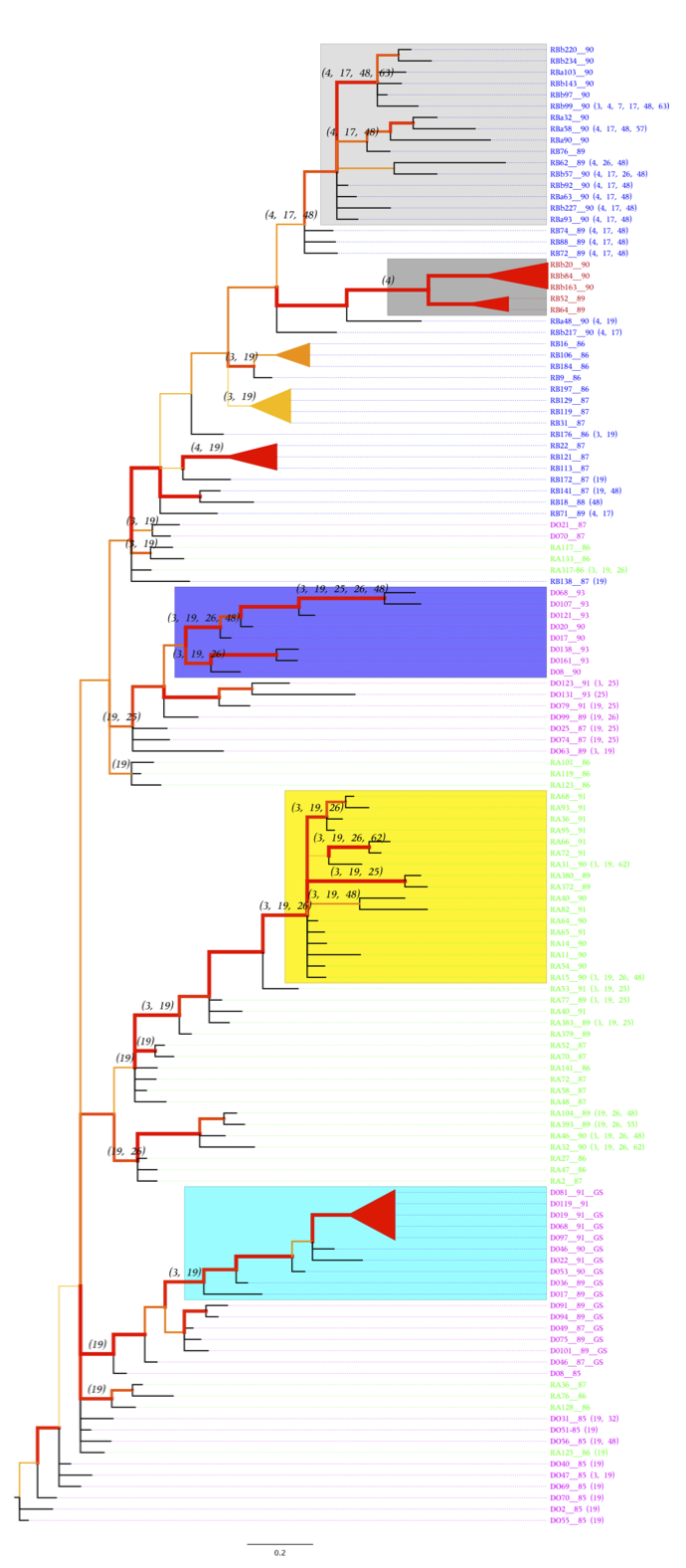

Supplement: Figure S3 — Glycosylation pattern of the V3 loop of HIV-1. Numbers above branches indicate glycosylated sites in the V3 region. The tree shows clones of within-host sequences of the blood donor DO (depicted in pink color) and the recipients RA (depicted in green) and RB (depicted in blue and red color for X4 isolates). Highlighted areas represent clusters of isolates obtained from the last time points in each individual. The statistical support of the tree is indicated by the colors of branches in a gradient scale from yellow to red, respectively indicating posterior probability of 0.5 to 0.99. Some branches were collapsed to facilitate visualization. (TIFF) [file pone.0039776.s003.tiff]

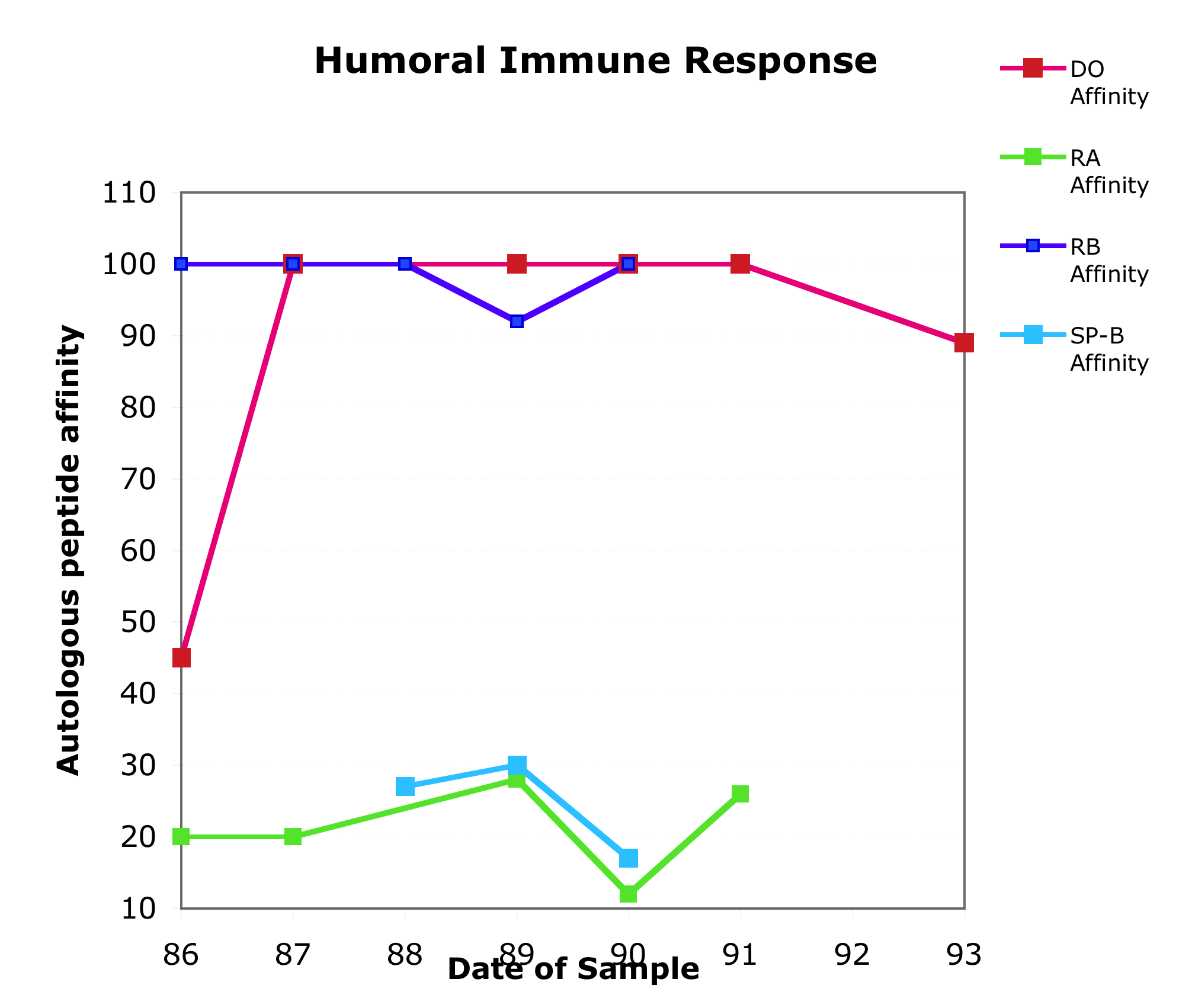

Supplement: Figure S4 — Antibody affinity by autologous synthetic peptides. The humoral immunity of each patient was measured by the affinity of their antibody to recognize peptides based on consensus sequences of virus isolated in distinct time points. Affinity was measured independently in each individual in distinct time points. (TIFF) [file pone.0039776.s004.tiff]

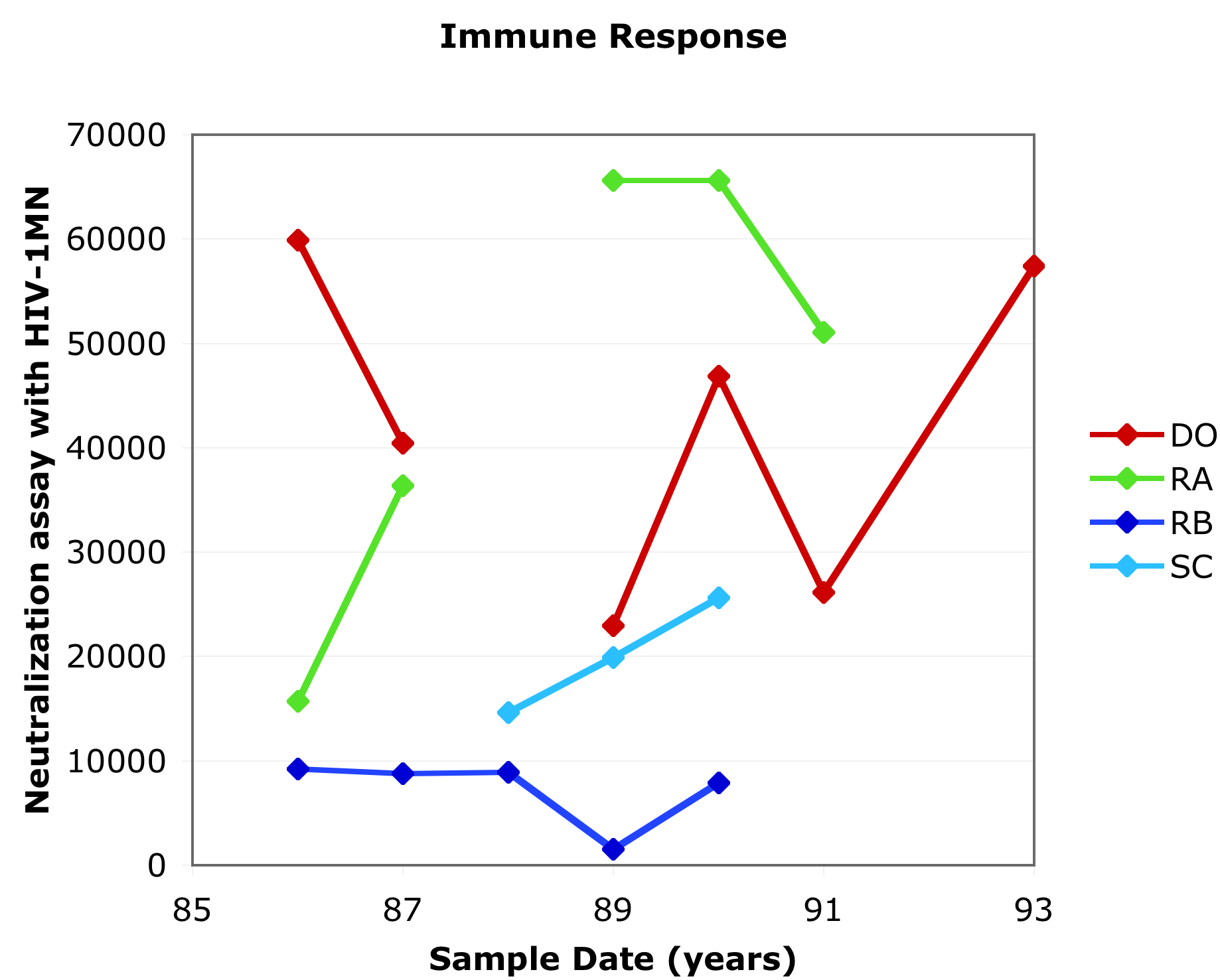

Supplement: Figure S5 — Neutralization of the heterologous MN strain of HIV-1. Humoral immune response against the MN strain of HIV-1 was measured in the plasma of patients during the over the infection time. Each line represent one individual and points are the measured immune response detected in each year (x-axis). (TIFF) [file pone.0039776.s005.tiff]

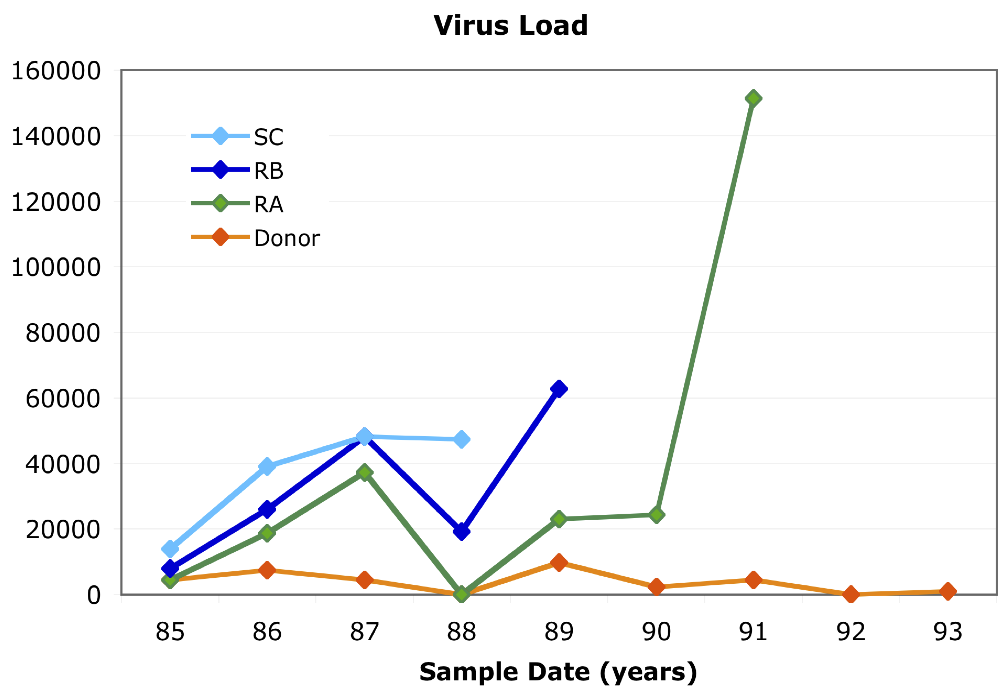

Supplement: Figure S6 — Virus load of each patient during infection time. The x-axis represents the sampling time of the study. Y-axis depicts the amount of viruses measured in RNA copies per ml of plasma. (TIFF) [file pone.0039776.s006.tiff]
